# Green synthesis of silver nanoparticles using guava leaves: an effective strategy to control chilli fruit rot disease

**DOI:** 10.1186/s12870-025-06528-4

**Published:** 2025-04-21

**Authors:** Saba Mazhar, Sajjad Hyder, Babar Shahzad Khan, Amjad Shahzad Gondal, Raees Ahmed, Iqra, Mudassir Iqbal

**Affiliations:** 1 Department of Botany, Faculty of Natural Sciences, Government College Women University, Sialkot, Pakistan; 2 Department of Physics, Faculty of Natural Sciences, Government College Women University, Sialkot, Pakistan; 3 Department of Plant Pathology, Bahauddin Zakariya University, Multan, Pakistan; 4 Department of Plant Pathology, University of Poonch, Rawalakot, AJK Pakistan; 5 Department of Plant Protection Biology, Swedish University of Agricultural Sciences, Lomma, Sweden

**Keywords:** Ag-NPs, Anthracnose, Antifungal activity, Chilli rot, Leaf extract

## Abstract

**Background:**

Anthracnose, caused by *Colletotrichum capsici*, is a significant fungal disease affecting chilli crops, leading to yield losses of 10–25%. Traditional control methods, primarily chemical fungicides, not only pose risks to the environment and soil health but also threaten public safety. In contrast, nanotechnology presents a promising eco-friendly alternative, leveraging the unique properties of nanoparticles, such as their small size and high surface-to-volume ratio, to effectively manage fungal infections with minimal environmental impact.

**Results:**

This study investigates the synthesis, characterization, and antifungal activity of silver nanoparticles (Ag-NPs) synthesized from guava leaf extract against chilli fruit rot. UV-Vis spectroscopy confirmed the synthesis of Ag-NPs with a peak absorption at 431 nm. X-ray diffraction (XRD) analysis revealed a crystalline structure with an average particle size of 42.5 nm, while scanning electron microscopy (SEM) showed spherical nanoparticles with sizes ranging from 30.5 nm to 50.3 nm across different samples. Fourier transform infrared spectroscopy (FTIR) identified functional groups involved in silver ion reduction. Zeta size analysis confirmed particle sizes of 500.1 nm, 1.0 nm, 62.4 nm, 262.8 nm, and 178.8 nm for samples S1 through S5, respectively. In antifungal assays, S1 at 50 ppm exhibited the highest mycelial growth inhibition (47.9%), with significant protective (87%) and curative (93%) effects. Additionally, in in-vitro leaflet assays, S1 demonstrated 86% inhibition of *C. capsici* at 50 ppm, highlighting its potential as an effective agent for managing chilli fruit rot.

**Conclusions:**

This study presents a rapid, eco-friendly method for synthesizing Ag-NPs using guava leaf extract, showing their potential in managing chilli fruit rot caused by *C. capsici*. The results highlight their effectiveness in both protective and curative applications, offering a sustainable alternative to chemical fungicides. Future research should focus on scaling up the synthesis process for industrial applications, exploring the long-term environmental impact, and assessing the broader applicability of Ag-NPs in managing other phytopathogenic diseases across various crops.

**Summary:**

Silver nanoparticles (Ag-NPs) synthesized from guava leaf extract effectively inhibit *Colletotrichum capsici*, with 86% antifungal activity at 50 ppm. This study highlights an eco-friendly, rapid synthesis method for Ag-NPs as a promising alternative to chemical fungicides in managing chilli fruit rot disease.

**Clinical trial number:**

Not applicable.

**Graphical Abstract:**

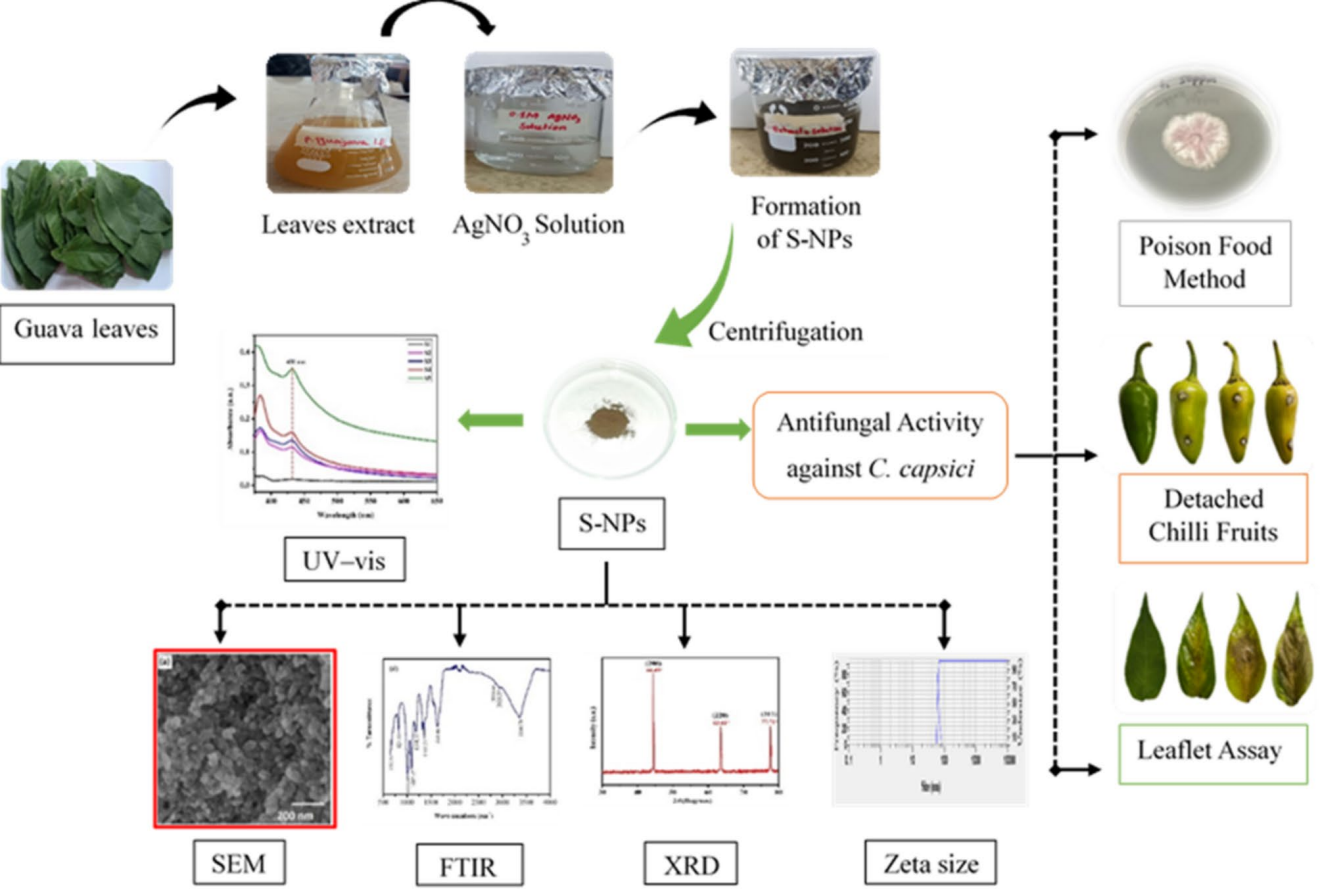

**Supplementary Information:**

The online version contains supplementary material available at 10.1186/s12870-025-06528-4.

## Introduction

Chilli (*Capsicum annuum* L.), a vital vegetable and lucrative crop, is cultivated globally and is a staple in Pakistan’s agriculture, contributing approximately 1.5% to the nation’s gross domestic production [1]. Despite an annual production of 103.7 thousand tonnes from 45.7 thousand hectares [2], chilli yields in Pakistan are severely affected by various pathogens and pests, with fungal diseases posing the most significant threat [3]. Among these, anthracnose or fruit rot caused by *Colletotrichum capsici* is particularly destructive, leading to annual yield losses of up to 84% [4]. Even minor lesions on chilli fruits significantly reduce their market value [5]. Current management strategies primarily rely on chemical fungicides. Despite their effectiveness, chemical fungicides have several drawbacks, including high costs, the development of resistant fungal strains, residual toxicity, and harmful effects on human health and the environment. Prolonged use of synthetic fungicides can lead to soil contamination, disruption of beneficial microbial communities, and bioaccumulation in the food chain, raising serious ecological and public health concerns [6]. Therefore, there is an urgent need for biocompatible, eco-friendly, cost-effective, and target-specific alternatives [7]. Recent research has explored various eco-friendly solutions, such as biological control [8], plant extracts [9], essential oils [10], and nanoparticles [11].

Among these nanoparticles, silver nanoparticles (Ag-NPs) have gained significant attention for their ability to manage phytopathogenic diseases. Although silver (Ag) itself possesses antimicrobial properties through its oxido-reduction forms [12], Ag-NPs offer several advantages that make them superior against pathogenic fungi. Due to their nanoscale size and high surface-area-to-volume ratio, Ag-NPs exhibit enhanced interaction with fungal cells, enabling better adhesion, membrane penetration, and intracellular disruption [13–19]. Their potent antimicrobial activity has been demonstrated against various pathogens, including bacteria, viruses, and fungi. Numerous synthesis methods for nanoparticles exist, such as chemical reduction, photochemical, thermal degradation, and biological approaches using natural resources like algae, fungi, bacteria, and plants [20–21]. Plant-based synthesis of Ag-NPs offers several advantages over other biological methods, including simplicity, scalability, and non-toxicity. This approach leverages plant extracts as natural reducing and stabilizing agents, eliminating the need for harmful chemicals typically used in chemical or microbial synthesis [22–23].

*Psidium guajava* L., commonly known as guava, is a plant from the Myrtaceae family used in both food and traditional medicine [24]. The leaf extract of *P. guajava* contains bioactive compounds such as polyphenols, flavonoids, and proteins, which can act as reductants to produce Ag-NPs rapidly at room temperature [25]. These compounds also possess antimicrobial properties due to their high polyphenolic content [26–27].

While many studies have documented the synthesis of Ag-NPs from plants such as *Alternanthera sessilis* [28], *Euphrasia officinalis* [29], and *Ziziphus spina-christi* [30], limited research has focused on their antifungal potential against specific agricultural pathogens. Addressing this gap, our study presents an eco-friendly method for synthesizing Ag-NPs using *P. guajava* leaf extract, whose unique phytochemicals provide targeted antifungal activity against *C. capsici*, the primary cause of chilli fruit rot. In this paper, we provide a detailed characterization of the Ag-NPs, assessing their particle size, structure, morphology, and demonstrate their antifungal efficacy across multiple concentrations, with both protective and curative effects. Our sustainable synthesis method, free from hazardous chemicals, offers a scalable, environmentally safe alternative for agricultural disease management, representing a promising advance in targeted nanoparticle applications.

## Materials and methods

### Plant and fungus materials

Fresh and healthy leaves of *P. guajava* were collected from cultivated trees growing on the campus of Government College Women University Sialkot (GCWUS), Pakistan (32°30’10.5"N, 74°31’50.0"E), and used for nanoparticle preparation. A virulent strain of *C. capsici* CC1 was obtained from the Department of Botany at GCWUS (https://gcwus.edu.pk/faculties/faculty-of-natural-science/botany/), Pakistan [31]. The strain was cultured on potato dextrose agar (PDA) medium (HCM050 HKM, China) and incubated at 28 ± 2ºC under dark conditions to ensure optimal growth and virulence [31].

### Green synthesis of Ag-NPs

The *P. guajava* leaf extract preparation was performed following [32]. Briefly, freshly collected leaves were washed with tap water, sterilized with 1% NaOCl for 2 min, and rinsed with distilled water. A total of 25 g of sterilized leaves were crushed using a sterile pestle and mortar, and then mixed with 100 mL of distilled water in a beaker covered with aluminum foil. The mixture was heated in a water bath at 80ºC for 15 min, cooled to room temperature, and filtered through Whatman No. 2 filter paper to remove debris. Silver nitrate (AgNO3) was dissolved in distilled water to prepare a 0.1 M solution, covered with aluminum foil, and shaken to ensure complete dissolution.

To synthesize Ag-NPs, the leaf extract and AgNO3 solutions were mixed in various ratios: 270:30, 240:60, 210:90, 180:120, and 150:150 (leaf extract). The mixtures were stirred at 1300 rpm for 30 min at room temperature, with a colour change from light yellow to dark brown indicating Ag-NPs formation. The mixtures were centrifuged at 4000 rpm for 10 min, the supernatants removed, and residues washed twice with distilled water. The precipitates were dried at 100ºC for 2 h, ground into a powder, and stored in Eppendorf tubes at room temperature for further characterization.

### Characterization of Ag-NPs

#### UV-visible spectroscopy

The synthesis of Ag-NPs was confirmed using UV-vis spectroscopy (Analytic Jena – Specord^®^210 Plus, Germany) at GCWUS, Pakistan, as described by [33]. In short, samples were prepared by adding 0.001 g of dried Ag-NPs to 10 mL of deionized water, sonicated for 15 min, and analysed for absorption spectra between 200 and 800 nm using WinASPECT Plus (ver. 4.2) software. The energy band gap was calculated using the Einstein photon energy equation [34].

### *X-ray diffraction analysis*

The structural properties of the Ag-NPs were studied using X-ray diffraction (XRD). To prepare the Ag-NPs sample for XRD analysis, the solvent was evaporated, and the nanoparticles were spread onto a glass slide to form a thin film. The crystal phase of the nanoparticles was identified using a Bruker D2 Phaser X-ray diffractometer (XRD, Bruker D2 Phaser, Berlin, Germany) equipped with Cu-Kα radiation (λ = 0.15406 nm). The diffractometer was operated over a scanning range of 30° to 80° with a step size of 0.05° [35]. The crystallite size was calculated using the Scherrer equation [34].

### *Fourier transform infrared spectroscopy (FTIR)*

FTIR spectroscopy was employed to identify the biomolecules in *P. guajava* leaves responsible for the reduction of silver ions and the stabilization of the Ag-NPs. The analysis was conducted using a Bruker Tensor II FTIR spectrometer (Germany). The FTIR spectra of the biosynthesized Ag-NPs were recorded in the wavelength range of 500–4000 cm⁻¹ with a resolution of 4 cm⁻¹. The samples were prepared by incorporating the Ag-NPs into potassium bromide (KBr) pellets. The functional groups and bonds present in the Ag-NPs were identified by analysing the distinct peaks observed in the FTIR spectra, as per the method described by [36].

### *Zeta size analysis*

The zeta size analysis of the Ag-NPs was conducted using a Zetasizer (Horiba SZ-100, ver. 2.40). The particle size of the Ag-NPs was measured by dynamic light scattering (DLS), which utilizes the scattering intensity of light to determine the size distribution of the nanoparticles in the suspension.

### *Scanning electron microscopy (SEM)*

The morphology and size of the Ag-NPs were analysed using a scanning electron microscope (SEM) with a TESCAN MIRA3 model, operating at 20 kV. A small aliquot of the Ag-NPs sample was deposited onto a carbon-coated copper grid and allowed to air-dry under a mercury lamp for 5 min to ensure proper adherence. The prepared samples were then examined under the SEM to capture high-resolution images. Particle size measurements and morphological assessments were conducted using ImageJ software (ver. 1.8) to analyse the SEM images.

### In-vitro evaluation of antifungal potential of synthesized Ag-NPs against *C. capsici*

#### Poison food technique

The in-vitro antifungal activity of Ag-NPs was evaluated using the poison food technique [37]. PDA Petri plates were prepared by incorporating 11% (w/v) Ag-NPs different concentrations into the PDA medium. To test antifungal efficacy, 6 mm plugs of actively growing *C. capsici* from 7 to 9 day-old cultures were aseptically transferred to the centre of the Ag-NPs -supplemented PDA plates. The plates were incubated at 28 ± 2 °C in the dark for 7 days. Control plates containing PDA without Ag-NPs were included for comparison. Each treatment was contained with three biological replicates. Fungal mycelial growth was measured in four directions, and the average diameter was calculated after 7 days. The percentage of mycelial growth inhibition was determined as described previously [38].

### *Evaluation of disease suppression potential of Ag-NPs against fruit rot disease on detached chilli fruits*

The efficacy of Ag-NPs in suppressing fruit rot disease was assessed using detached chilli fruits, following a modified protocol from [39]. Chilli fruits were initially sterilized by soaking in 1% sodium hypochlorite (NaOCl) for 2 min, followed by two rinses with distilled water. They were then air-dried on sterile filter paper in aseptic conditions and placed in plastic containers lined with two layers of tissue paper. For inoculation, the fruits were punctured at five points, each to a depth of 2 mm, using a sterile inoculating needle. An actively growing culture of *C. capsici* was used to inoculate these points. Ag-NPs at concentrations of 50 ppm, 100 ppm, 150 ppm, 250 ppm, 500 ppm, and 1000 ppm were tested. The control group received sterile distilled water. The chilli fruits were divided into two experimental groups: in the protective assay, Ag-NPs were applied by spraying 48 h prior to pathogen inoculation, while in the curative assay, Ag-NPs treatments were applied immediately after inoculation. All treated fruits were incubated at 27 ± 1 °C for 15 days before measuring lesion sizes. Each treatment was replicated three times, with four fruits per replicate.

### *Leaflet assay for Ag-NPs antifungal activities*

Fresh, healthy chilli leaves were collected from Malkhanwala, Pakistan. The leaves were first disinfected by following the protocol as described above. Post-disinfection, the leaves were treated with various concentrations of aqueous Ag-NPs solutions, specifically 50 ppm, 100 ppm, 150 ppm, 250 ppm, 500 ppm, and 1000 ppm. After application, the leaves were inoculated with fungal plugs of *C. capsici* derived from a 7-day-old culture. The inoculated leaves were placed in sterilized plastic boxes and incubated at 27 ± 1 °C for 7 days. Control leaves were treated with distilled water only. Each treatment was replicated three times, with four leaves per replicate. The development of necrotic lesions was assessed by measuring their diameters after 7 days.

### Statistical analysis

The in vitro antagonism assay data were analyzed using ANOVA with a general linear model approach in OriginPro (ver. 8.5) and Statistix (ver. 8.1). Pairwise comparisons were conducted using either the Tukey-Kramer method or Fisher’s least significant difference at 95% significance level.

## Results

### Synthesis of Ag-NPs

The biological synthesis of Ag-NPs was carried out using an aqueous extract of *P. guajava* leaves, which served as both a reducing and stabilizing agent. The bioactive compounds in the guava leaf extract facilitated the reduction of Ag⁺ ions to Ag⁰ during the nanoparticle formation process. To prepare the reaction mixture, various volumes of 0.1 M AgNO₃ and the guava leaf extract were combined. Continuous stirring at room temperature resulted in a colour change from light yellow to dark brown within 30 min, indicating the successful formation of Ag-NPs.

### Characterization of Ag-NPs

**Fig. 1:**
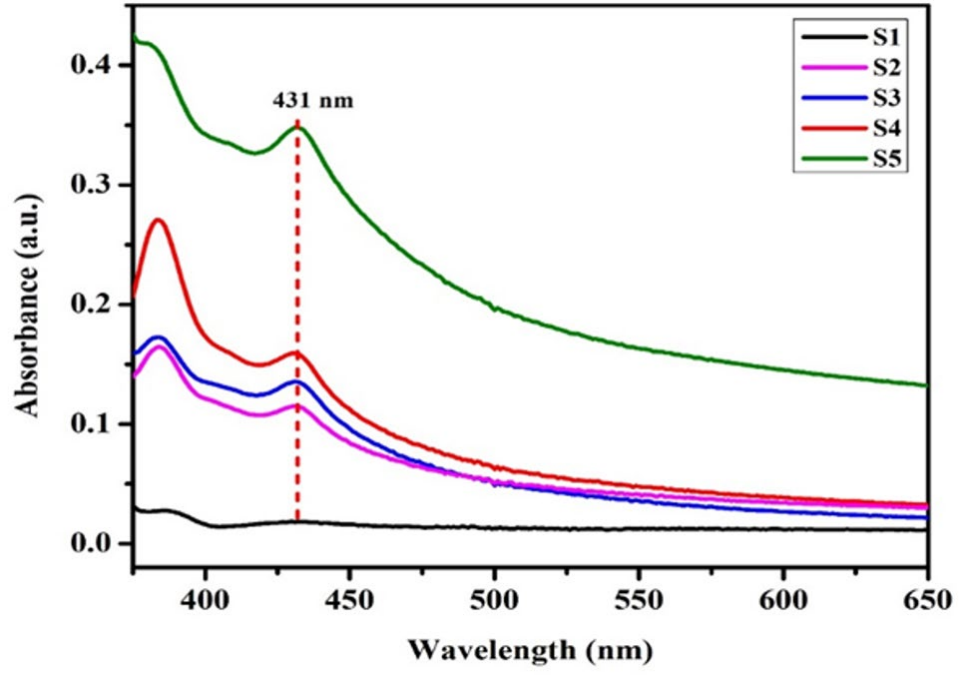
The UV-Vis absorbance spectrum of silver nanoparticles (S-NPs) synthesized using *Psidium guajava* leaf extract, showing distinct absorbance peaks within the wavelength range of 200–800 nm

The synthesis of Ag-NPs was confirmed using UV–Visible spectroscopy, with spectra collected over a range of 200–800 nm. A prominent absorbance peak at 431 nm, attributed to surface plasmon resonance (SPR), was observed in all samples (S1 to S5), indicating the successful formation of Ag-NPs (Fig. 1). The energy bandgap of the synthesized Ag-NPs was calculated using the Einstein photon energy equation, based on the UV–Visible absorption spectrum. The calculated bandgap for all samples was 2.87 eV.

### XRD analysis

**Fig. 2:**
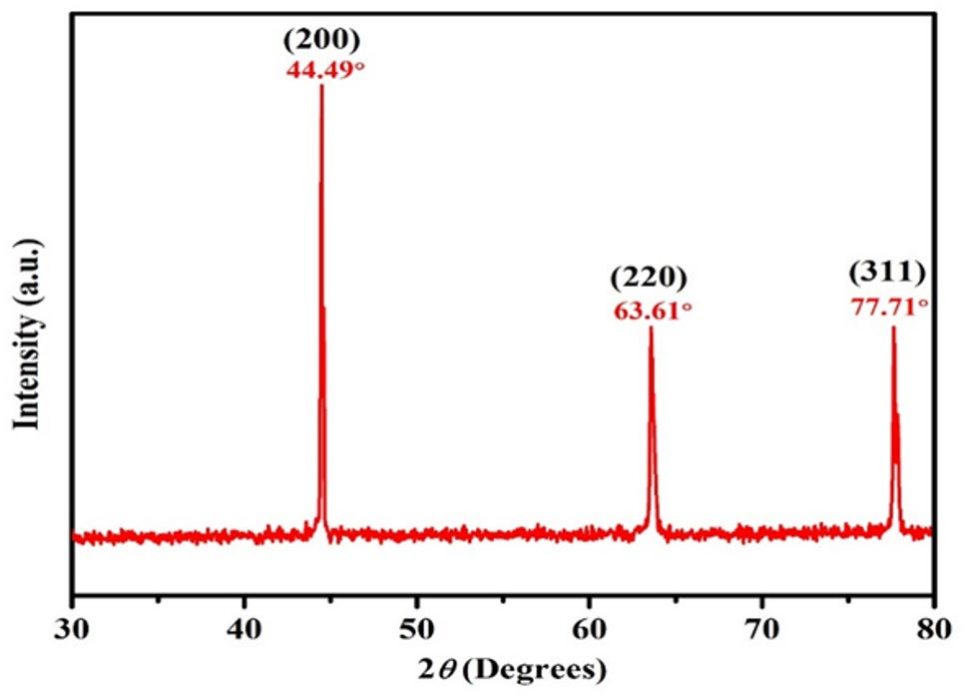
X-ray diffraction (XRD) pattern of silver nanoparticles (S-NPs) synthesized using *Psidium guajava* leaf extract. The diffraction peaks at specific 2θ values indicate the crystalline nature and face-cantered cubic (FCC) structure of the S-NPs, consistent with the Joint Committee on Powder Diffraction Standards (JCPDS) card No. 87–719

The XRD spectrum, shown in Fig. 2, was used to determine the structural and crystalline properties of the biosynthesized Ag-NPs. Diffraction data were recorded within a range of 30° to 80°. The XRD pattern displayed three prominent Bragg diffraction peaks at 2θ values of 44.49°, 63.61°, and 77.71°, which correspond to the 200, 220, and 311 lattice planes of a face-centred cubic (FCC) crystal structure of Ag-NPs. These peaks confirm the crystalline nature of the synthesized Ag-NPs, in agreement with the Joint Committee on Powder Diffraction Standards (JCPDS) standard card No. 87–719. The average particle size was calculated using the Debye-Scherrer equation, as presented in supplementary Table S1.

### FTIR analysis

FTIR analysis was performed to identify the functional groups present in the synthesized Ag-NPs. The FTIR spectra of the five samples (S1 to S5) exhibited multiple peaks, each corresponding to different functional groups, confirming their presence in the synthesized nanoparticles.

#### FTIR analysis of S1

**Fig. 3:**
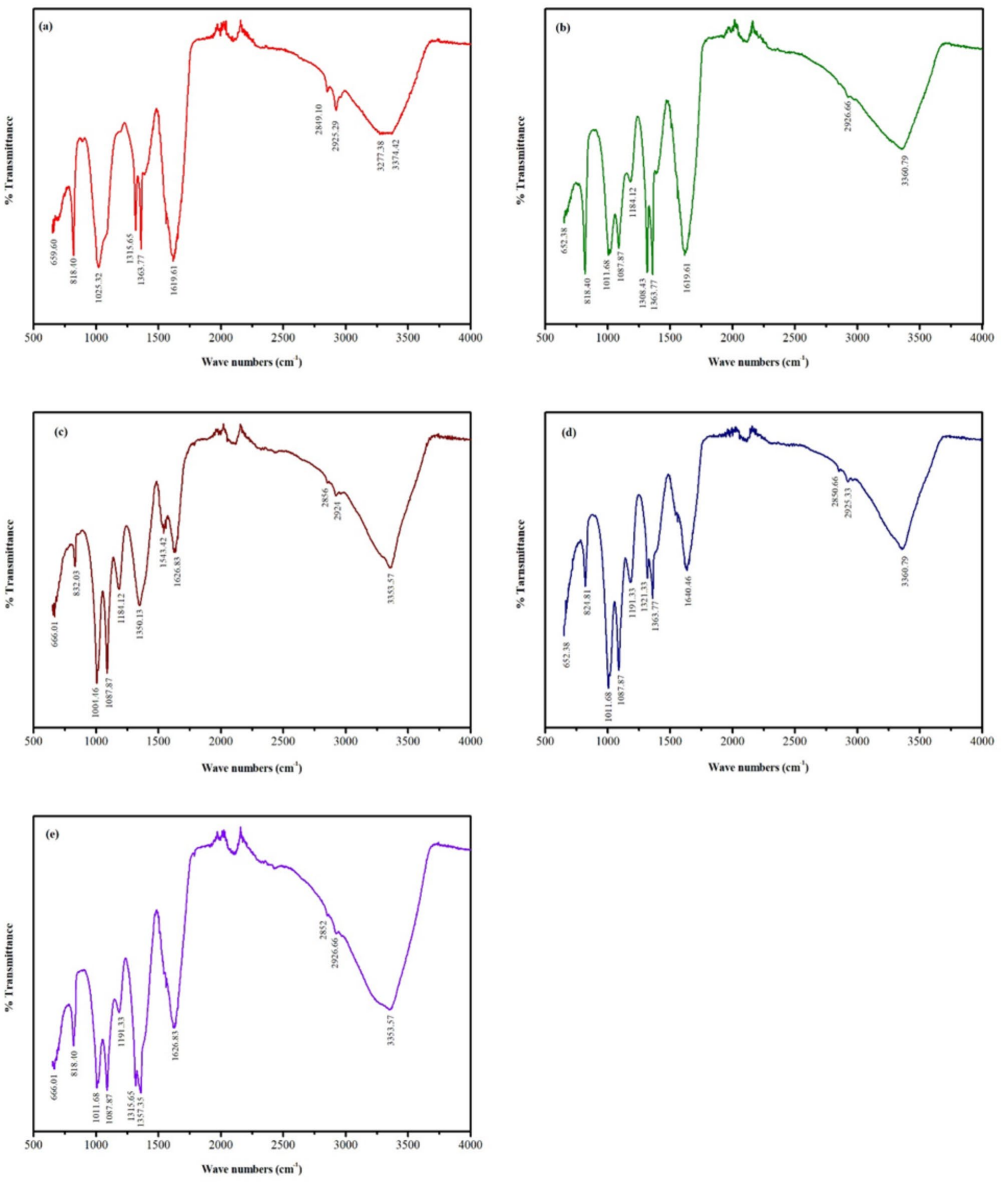
Fourier transform infrared spectroscopy (FTIR) spectrum of biosynthesized silver nanoparticles (S-NPs) from *Psidium guajava* leaf extract. (**a**) FTIR analysis of S1 reveals characteristic peaks corresponding to functional groups such as hydroxyl (O-H), carbonyl (C = O), and amine (N-H) groups, indicating their involvement in the reduction and stabilization of S-NPs. (**b**) S2 shows similar functional groups, including hydroxyl (O-H), carboxyl (C-OH), and aromatic compounds (C = C), which play a crucial role in the nanoparticle synthesis process. (**c**) S3 displays absorption bands corresponding to O-H, C = O, and C = C groups, confirming the participation of phytochemicals in the formation of S-NPs. (**d**) S4 highlights the presence of functional groups such as hydroxyl (O-H), amine (N-H), and carboxyl (C-OH), further supporting their role in the synthesis and stabilization of the nanoparticles. (**e**) S5 identifies characteristic peaks for various functional groups, including hydroxyl (O-H), carbonyl (C = O), and aromatic compounds (C = C), facilitating the reduction and capping of S-NPs. The FTIR spectra across all samples demonstrate the involvement of these functional groups in the green synthesis of S-NPs

FTIR analysis of S1 identified ten major peaks (Fig. 3a, Table S2), indicating the presence of various functional groups. The peak at 659.60 cm⁻¹ corresponded to C-C/C-H stretching vibrations of aromatic benzene groups, while the peak at 818.40 cm⁻¹ was attributed to C-H, C-O, and C-S stretching in aromatic rings. The band at 1025.32 cm⁻¹ represented O-H, C-N, and C-O stretching, characteristic of phenols, alcohols, esters, and ethers.

Peaks at 1315.65 cm⁻¹ and 1363.77 cm⁻¹ were associated with C═C and O-H stretching in alkenes and alcohols/phenols, along with C-H/C-O bending and N-O/N═O stretching in alkyl ketones and nitric groups. The 1619.61 cm⁻¹ peak indicated C═O/C═C stretching and N-H bending, suggesting the presence of carboxylic acids, ketones, alkenes, aromatic rings, and carbonyl amides.

Further, peaks at 2849.10 cm⁻¹ and 2925.29 cm⁻¹ corresponded to symmetric and asymmetric C-H stretching in alkanes, as well as O-H stretching in phenols and alcohols. Finally, the broad bands at 3277.38 cm⁻¹ and 3374.42 cm⁻¹ were attributed to O-H, C-H, and N-H stretching, indicative of phenols, alcohols, alkynes, and aliphatic amines (Fig. 3a, Table S2).

#### FTIR analysis of S2

FTIR analysis of S2 revealed ten major peaks, indicating the presence of various functional groups (Fig. 3b, Table S3). The peak at 652.38 cm⁻¹ corresponded to C-C/C-H stretching of aromatic benzene groups, while the 818.40 cm⁻¹ peak was associated with C-H, C-O, and C-S stretching in aromatic rings. Peaks at 1011.68 cm⁻¹ and 1087.87 cm⁻¹ were linked to C═C bending in alkenes and O-H/C-N/C-O stretching in phenols, alcohols, esters, and ethers, whereas the 1184.12 cm⁻¹ peak was attributed to O-H/C-N stretching and asymmetric C-O stretching in alkyl amines, alcohols, esters, and ethers.

Further, peaks at 1308.43 cm⁻¹ and 1363.77 cm⁻¹ indicated C═C and O-H stretching in alkenes and alcohols/phenols, along with C-H/C-O bending and N-O/N═O stretching in alkyl ketones and nitric groups. The peak at 1619.61 cm⁻¹ corresponded to C═O/C═C stretching and N-H bending, suggesting the presence of carboxylic acids, ketones, alkenes, aromatic rings, and carbonyl amides. The band at 2926.66 cm⁻¹ was linked to O-H stretching and symmetric/asymmetric C-H stretching in phenols, alcohols, and alkanes, while the broad peak at 3360.79 cm⁻¹ was associated with O-H, C-H, and N-H stretching in phenols, alcohols, alkynes, and aliphatic amines.

#### FTIR analysis of S3

FTIR analysis of S3 revealed 11 major peaks, indicating the presence of various functional groups (Fig. 3C, Table S4). The peak at 666.01 cm⁻¹ corresponded to C-C/C-H stretching of aromatic benzene groups, while peaks at 832.03 cm⁻¹ and 1004.46 cm⁻¹/1087.87 cm⁻¹ were associated with C-H, C-O, and C-S stretching in aromatic rings, as well as C═C bending in alkenes and O-H/C-N/C-O stretching in phenols, alcohols, esters, and ethers. The 1184.12 cm⁻¹ peak was attributed to O-H/C-N stretching and asymmetric C-O stretching in alkyl amines, alcohols, esters, and ethers.

Peaks at 1350.13 cm⁻¹ and 1363.77 cm⁻¹ indicated C═C and O-H stretching in alkenes and alcohols/phenols, along with C-H/C-O bending and N-O/N═O stretching in alkyl ketones and nitric groups. The 1543.42 cm⁻¹ peak corresponded to protein stretching vibrations in amides, while the 1626.83 cm⁻¹ peak reflected C═O/C═C stretching and N-H bending, characteristic of carboxylic acids, ketones, alkenes, aromatic rings, and carbonyl amides.

The bands at 2856 cm⁻¹ and 2924 cm⁻¹ were associated with symmetric and asymmetric C-H stretching in alkanes, phenols, and alcohols. Lastly, the broad peak at 3353.57 cm⁻¹ was attributed to O-H, C-H, and N-H stretching in phenols, alcohols, alkynes, and aliphatic amines.

#### FTIR analysis of S4

FTIR analysis of S4 identified 11 major peaks, indicating the presence of various functional groups (Fig. 3D, Table S5). The peak at 652.38 cm⁻¹ corresponded to C-C/C-H stretching of aromatic benzene groups, while peaks at 824.81 cm⁻¹ and 1011.68 cm⁻¹/1087.87 cm⁻¹ were associated with C-H, C-O, and C-S stretching in aromatic rings, along with C═C bending in alkenes and O-H/C-N/C-O stretching in phenols, alcohols, esters, and ethers. The 1191.33 cm⁻¹ peak was attributed to O-H/C-N stretching and asymmetric C-O stretching in alkyl amines, alcohols, esters, and ethers.

Peaks at 1321.33 cm⁻¹ and 1363.77 cm⁻¹ corresponded to C═C and O-H stretching in alkenes and alcohols/phenols, as well as C-H/C-O bending and N-O/N═O stretching in alkyl ketones and nitric groups. The 1640.46 cm⁻¹ peak reflected C═O/C═C stretching and N-H bending, characteristic of carboxylic acids, ketones, alkenes, aromatic rings, and carbonyl amides.

The bands at 2850.66 cm⁻¹ and 2925.33 cm⁻¹ were linked to symmetric and asymmetric C-H stretching in alkanes, phenols, and alcohols. Lastly, the broad peak at 3360.79 cm⁻¹ was attributed to O-H, C-H, and N-H stretching in phenols, alcohols, alkynes, and aliphatic amines.

#### FTIR analysis of S5

FTIR analysis of S5 identified 11 major peaks, indicating the presence of various functional groups (Fig. 3E, Table S6). The peak at 666.01 cm⁻¹ corresponded to C-C/C-H stretching of aromatic benzene groups, while the 818.40 cm⁻¹ peak was associated with C-H, C-O, and C-S stretching in aromatic rings. Peaks at 1011.68 cm⁻¹ and 1087.87 cm⁻¹ were attributed to C═C bending in alkenes and O-H/C-N/C-O stretching in phenols, alcohols, esters, and ethers. The 1191.33 cm⁻¹ peak reflected O-H/C-N stretching and asymmetric C-O stretching in alkyl amines, alcohols, esters, and ethers.

Peaks at 1315.65 cm⁻¹ and 1357.35 cm⁻¹ indicated C═C and O-H stretching in alkenes and alcohols/phenols, along with C-H/C-O bending and N-O/N═O stretching in alkyl ketones and nitric groups. The 1626.83 cm⁻¹ peak was linked to C═O/C═C stretching and N-H bending, characteristic of carboxylic acids, ketones, alkenes, aromatic rings, and carbonyl amides.

The bands at 2852 cm⁻¹ and 2926.66 cm⁻¹ corresponded to symmetric and asymmetric C-H stretching in alkanes, phenols, and alcohols. Lastly, the broad peak at 3353.57 cm⁻¹ was attributed to O-H, C-H, and N-H stretching in phenols, alcohols, alkynes, and aliphatic amines.

#### Zeta size analysis

**Fig. 4:**
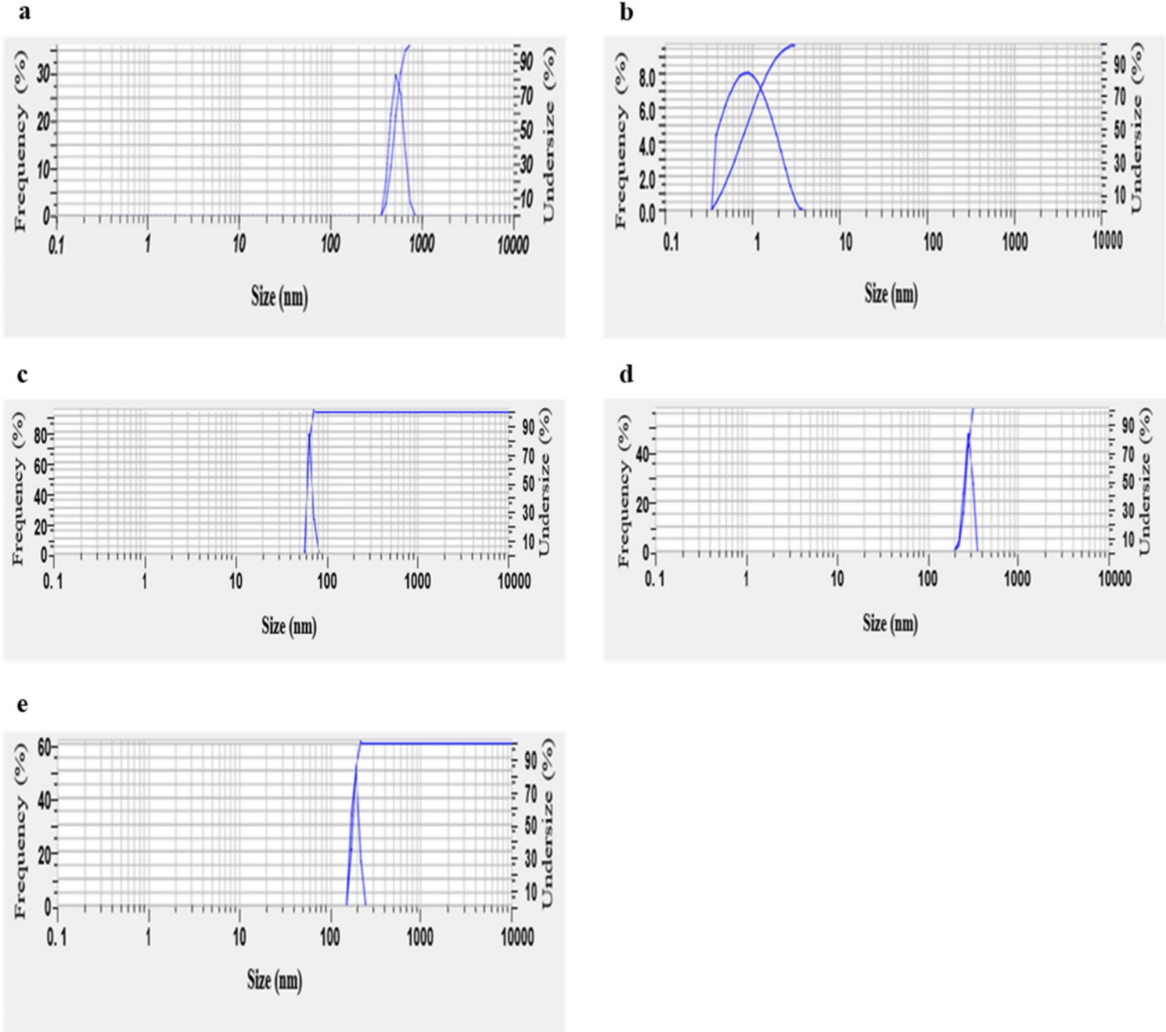
Zeta size analysis curve of biosynthesized silver nanoparticles (S-NPs) from *Psidium guajava* leaf extract. (**a**) Zeta size analysis of S1 shows a particle size distribution with an average diameter indicating the uniformity and stability of the synthesized nanoparticles. (**b**) Zeta size analysis of S2 (**c**) Zeta size analysis of S3 (**d**) Zeta size analysis of S4 (**e**) Zeta size analysis of S5. The zeta size analysis across all samples confirms the successful production of S-NPs with desirable size characteristics, essential for their potential applications in antifungal treatments

The particle size distribution of the synthesized Ag-NPs varied significantly across different samples, influencing their potential applications (Fig. 4). S1 exhibited the largest average particle size at 500.1 nm, which may be beneficial for applications requiring larger nanoparticles. In contrast, S2 had an exceptionally small average size of 1.0 nm, suggesting its classification within the quantum dot range, making it advantageous for applications demanding high surface area or quantum effects. S3, with an average size of 62.4 nm, fell within a medium nanoparticle range, making it suitable for various industrial applications where moderate particle sizes are preferred. S4 and S5 had average sizes of 262.8 nm and 178.8 nm, respectively, positioning them within the intermediate-to-large nanoparticle category. These variations in particle size could significantly influence the physicochemical properties of the nanoparticles, as optical and catalytic behaviours tend to differ between smaller and larger particles.

### SEM analysis

**Fig. 5:**
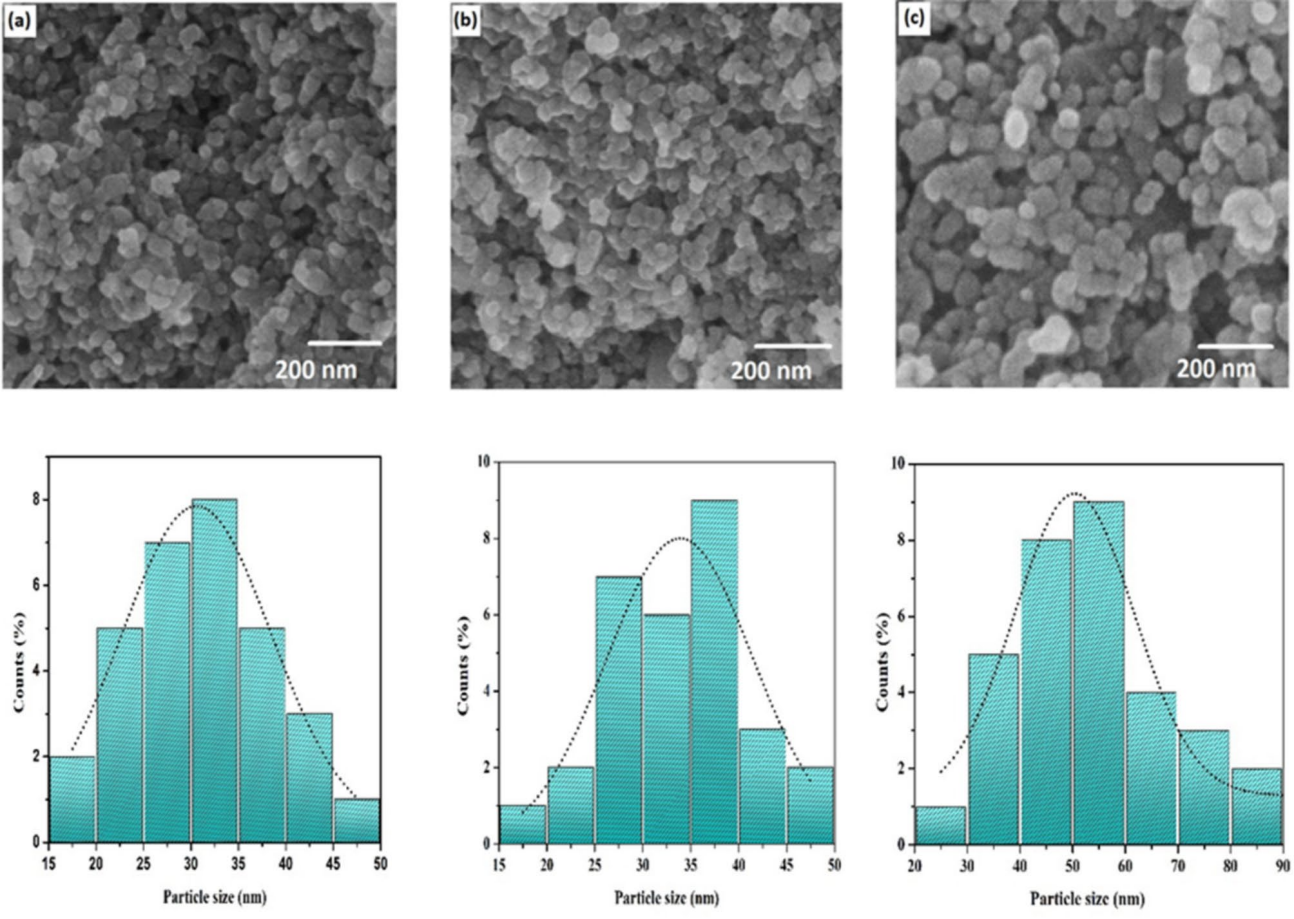
Scanning electron microscopy (SEM) images and particle size distribution histograms of silver nanoparticles (S-NPs) synthesized using *Psidium guajava* leaf extract. (**a**) SEM image of S1 displays spherical nanoparticles with a relatively uniform size and shape. (**b**) S3 reveals spherical particles with a similar morphology, indicating consistent synthesis across different batches. (**c**) S5 shows well-defined spherical nanoparticles, further supporting the reproducibility of the synthesis method. The accompanying histograms depict the particle size distribution of the S-NPs at a 200 nm scale: (**a**) S1, (**b**) S3, and (**c**) S5. Each histogram illustrates the frequency of particle sizes within the specified range, demonstrating a concentration of nanoparticles around the mean size, which aligns with the visual observations from the SEM images

SEM analysis was performed to examine the morphology and size distribution of the synthesized Ag-NPs. The SEM micrographs revealed that the Ag-NPs predominantly exhibit a spherical shape across all samples. Specifically, the average diameters of the Ag-NPs were 30.5 nm for S1, 33.8 nm for S3, and 50.3 nm for S5, as shown in Fig. 5a and b, and 5c, respectively. These measurements indicate a relatively uniform size distribution within each sample, although notable differences in size were observed between them. The particle size histograms, also presented at a 200 nm scale (Fig. 5a, b, and c), further illustrate the size distribution and confirm the predominance of spherical particles. These histograms provide detailed insights into the variation in particle size within each sample. The consistent spherical shape and varying sizes across the different Ag-NPs samples emphasize the potential for tailoring particle characteristics for specific applications based on size and uniformity.

### Effect of Ag-NPs on mycelial growth inhibition of *C. capsici*

**Fig. 6:**
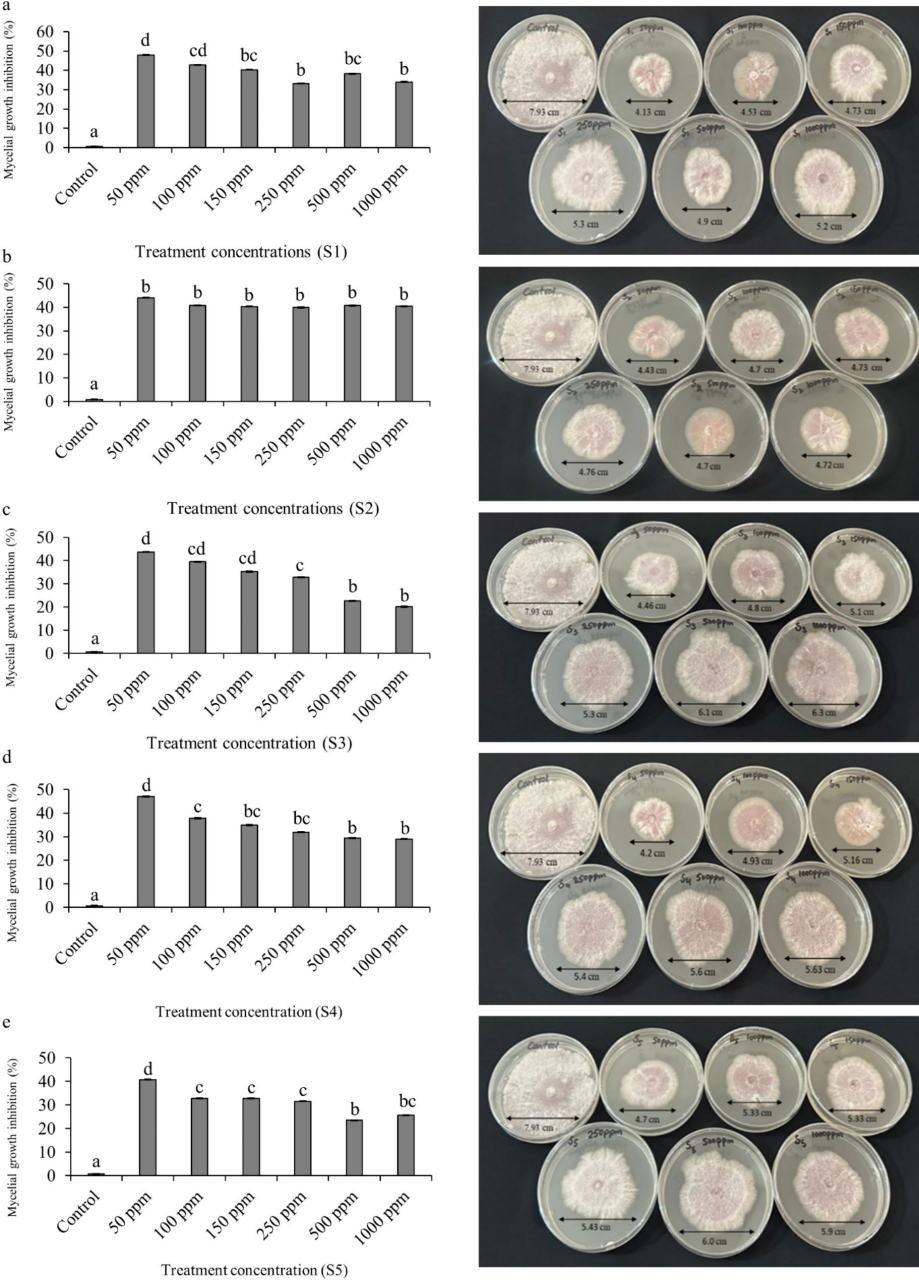
In-vitro antifungal activity of silver nanoparticles (S-NPs) synthesized from *Psidium guajava* leaves extract, demonstrating mycelial growth inhibition (%) against *Colletotrichum capsici*. The panels show the inhibition data for different S-NP samples at various concentrations: (**a**) S1, (**b**) S2, (**c**) S3, (**d**) S4, and (**e**) S5. Each pair of panels represents the inhibition results at different concentrations (50, 100, 150, 250, 500, and 1000 ppm) compared to the control. The significant differences in means (*p* ≤ 0.05) determined by least significant difference (LSD) test. Mean values sharing the same letter are not significantly different, whereas different letters indicate significant differences among the treatments

The antifungal efficacy of Ag-NPs synthesized from plant extracts was evaluated against *C. capsici* using the poisoned food technique. This method involved incorporating various concentrations of Ag-NPs (50 ppm, 100 ppm, 150 ppm, 250 ppm, 500 ppm, and 1000 ppm) into the growth medium and assessing their impact on fungal mycelial growth, as observed on Petri dishes. Among the five Ag-NPs samples tested, S1 exhibited the highest antifungal activity, with a maximum reduction in mycelial growth of 47.9% at a concentration of 50 ppm. S4 followed closely with a 47% reduction, while S2, S3, and S5 showed reductions of 44.1%, 43.7%, and 40.7%, respectively (Fig. 6). These results were compared to the control, which showed no inhibition. All concentrations of all Ag-NPs samples significantly inhibited mycelial growth compared to the control, highlighting the potential of these Ag-NPs as effective agents for controlling *C. capsici*.

### In-vitro evaluation of antifungal activity of Ag-NPs in detached Chilli fruits

**Fig. 7:**
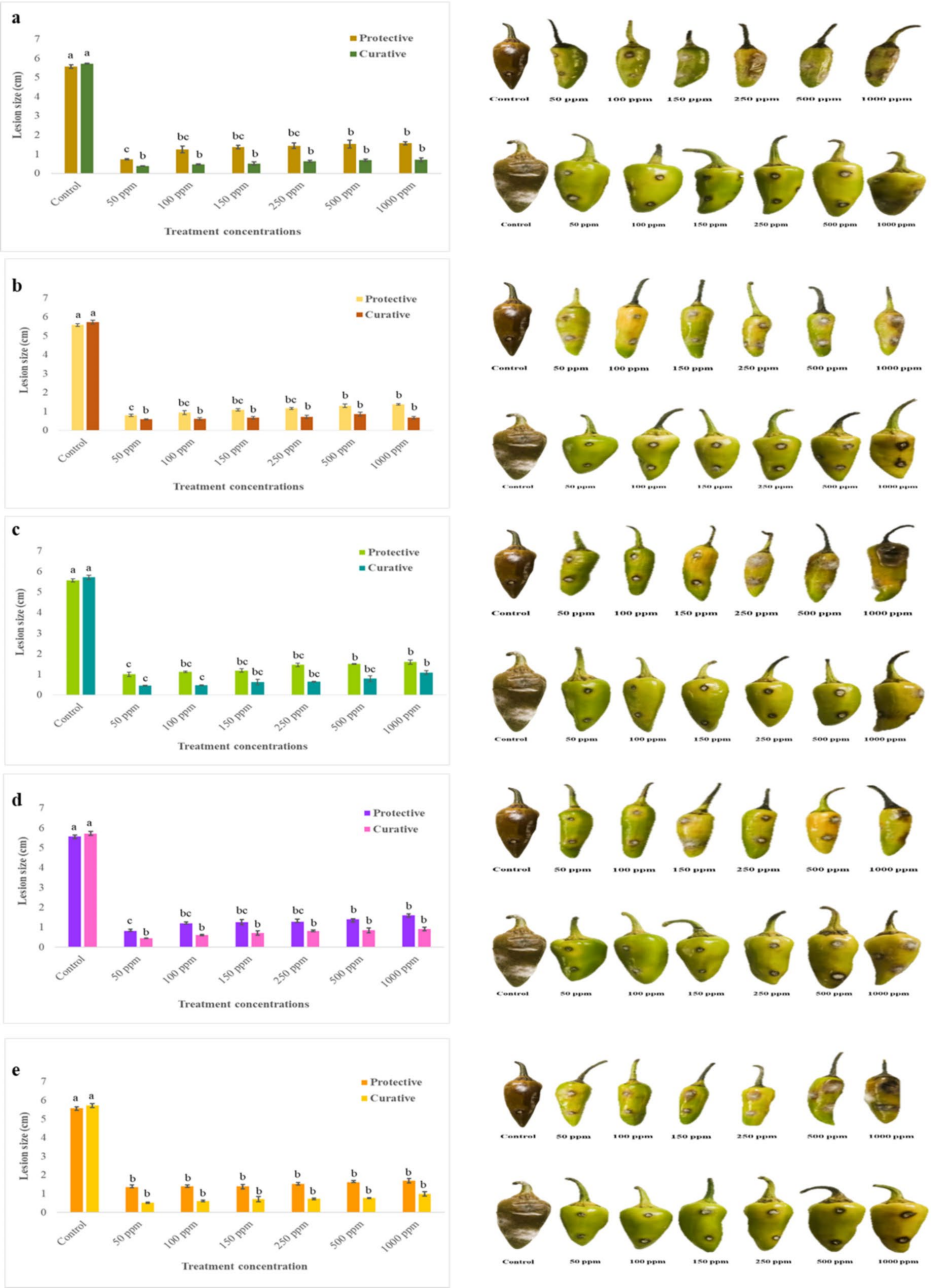
In-vitro efficacy of silver nanoparticles (S-NPs) at various concentrations in suppressing chilli anthracnose disease caused by *Colletotrichum capsici* using detached chilli fruits assay. The panels depict results for different S-NP samples: Panel a for S1, b for S2, c, for S3, d, for S4, and e, for S5. The significant differences in means (*p* ≤ 0.05) determined by least significant difference (LSD) test. Mean values sharing the same letter are not significantly different, whereas different letters indicate significant differences among the treatments

The antifungal efficacy of Ag-NPs was evaluated using detached chilli fruits, with various concentrations (50, 100, 150, 250, 500, and 1000 ppm) applied against *C. capsici*. Both protective and curative treatments were employed to assess the ability of Ag-NPs to manage fungal infection. In the protective assay, where Ag-NPs were applied before infection, all samples significantly reduced lesion sizes compared to the untreated control. At 50 ppm, S1 demonstrated the greatest reduction in lesion size to 0.73 cm, followed by S2, S3, S4, and S5, with reductions to 0.80 cm, 0.995 cm, 0.82 cm, and 1.35 cm, respectively (Fig. 7). In the curative treatment, where Ag-NPs were applied after infection, S1 achieved the most significant reduction with a lesion size of 0.385 cm, while S2, S3, S4, and S5 reduced lesion sizes to 0.58 cm, 0.455 cm, 0.44 cm, and 0.51 cm, respectively (Fig. 7). These results demonstrate that Ag-NPs exhibit substantial antifungal activity, effectively inhibiting the growth of *C. capsici* on chilli fruits in both preventive and curative applications. The findings underscore the potential of Ag-NPs as effective agents for managing *C. capsici* infections in practical settings.

### In-vitro antifungal activity of Ag-NPs using the leaflet assay technique

**Fig. 8:**
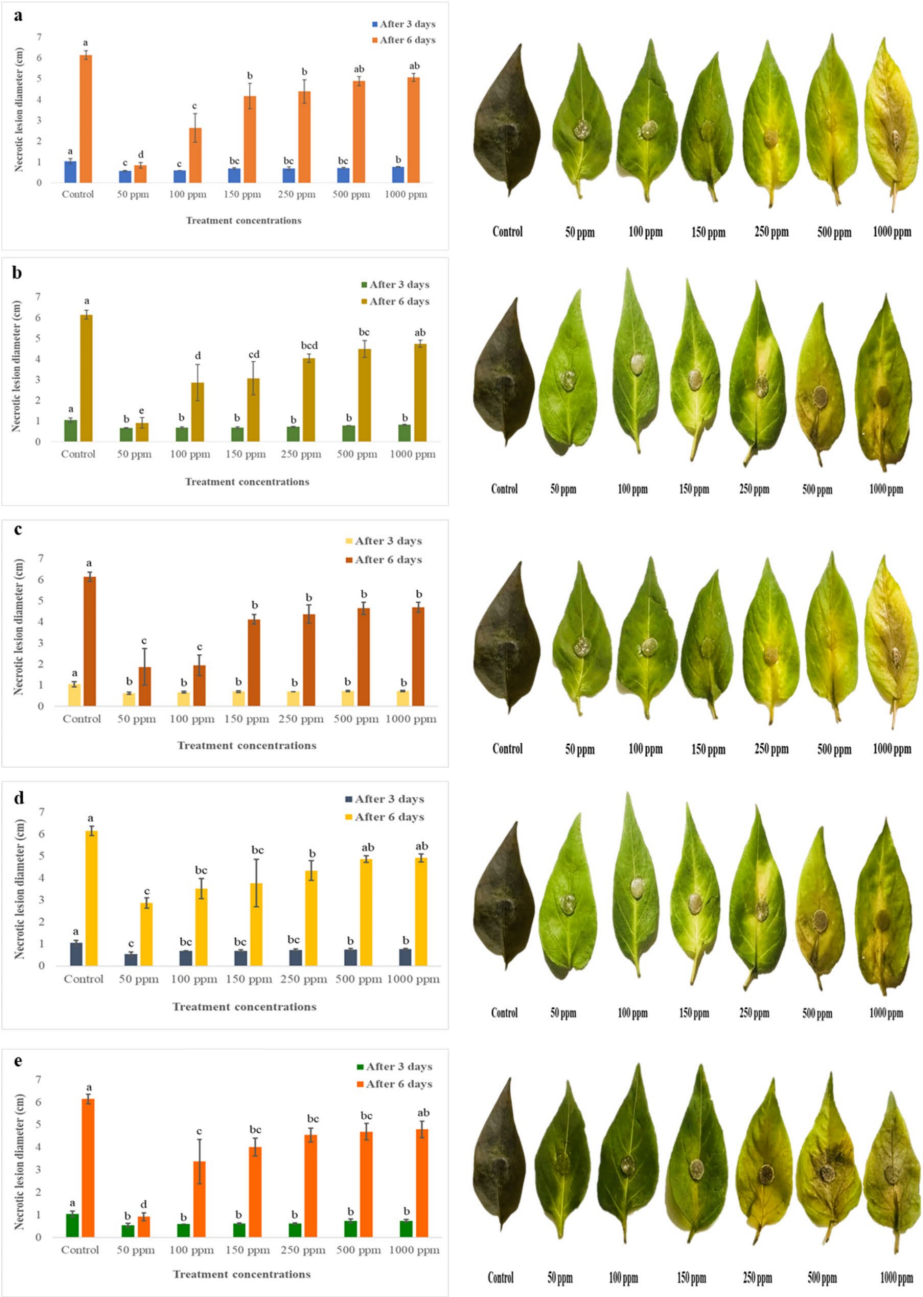
In-vitro antifungal activity of silver nanoparticles (S-NPs) at different concentration levels (50, 100, 150, 250, 500, and 1000 ppm) against *Colletotrichum capsici* using the detached leaflet assay. The panels show results for different S-NP samples: a, for S1; b, for S2; c for S3; d, for S4; and e, for S5. Each concentration demonstrates the varying degrees of mycelial growth inhibition, highlighting the effectiveness of the S-NPs in controlling fungal infection at different dosages. Mean values sharing the same letter are not significantly different, whereas different letters indicate significant differences among the treatments

The leaflet assay was performed on one-month-old chilli leaves infected with *C. capsici* and treated with varying concentrations (50, 100, 150, 250, 500, and 1000 ppm) of Ag-NPs from all five samples. The results revealed that antifungal efficacy was highest at lower concentrations of Ag-NPs. In the absence of Ag-NPs treatment, the leaves showed extensive necrotic lesions and wilting (Fig. 8). Among the tested concentrations, 50 ppm of Ag-NPs demonstrated the most significant reduction in lesion diameter, with S1 showing a decrease to 0.85 cm (86% reduction), followed by S2 at 0.92 cm (85%), S5 at 0.925 cm (84.9%), S3 at 1.875 cm (69.5%), and S4 at 2.875 cm (53.2%) compared to the untreated control, which had a lesion diameter of 6.15 cm (no reduction). In contrast, the lowest inhibition was observed at 1000 ppm, where lesion diameters were 5.07 cm (17.5%) for S1, 4.75 cm (22.7%) for S2, 4.7 cm (23.5%) for S3, 4.925 cm (19.9%) for S4, and 4.8 cm (21.9%) for S5 (Fig. 8). These findings highlight the potent antifungal activity of Ag-NPs, especially at lower concentrations, demonstrating their potential to effectively control fungal infections on chilli leaves.

## Discussion

Chilli is an economically significant crop grown worldwide, faces various biotic challenges, with anthracnose caused by *C. capsici* being among the most destructive, significantly reducing productivity and quality [40–42]. Nanotechnology offers a promising, innovative approach to plant protection, particularly in managing fungal diseases.

The synthesis of Ag-NPs was confirmed by a colour change from yellow to dark brown, indicating the excitation of surface plasmon resonance (SPR), consistent with previous reports [43, 44]. Similar syntheses using various plant extracts have demonstrated the visual formation of Ag-NPs through colour changes [14, 45–49]. The UV-Vis spectrophotometer analysis showed an absorption peak at 431 nm, consistent with SPR bands observed in previous studies [32, 50–52]. The energy bandgap of 2.87 eV calculated in this study aligns with earlier research findings [46, 53]. XRD analysis confirmed the face-centred cubic (FCC) structure of the biosynthesized Ag-NPs, with peaks corresponding to crystallographic planes, verifying their crystalline nature, consistent with the JCPDS (card No. 87–719) and previous studies [14, 17, 54–56].

FTIR spectroscopy identified functional groups involved in Ag-NPs synthesis, including hydroxyl, carboxyl, aromatic compounds, and amines, supporting the role of plant metabolites as reducing and stabilizing agents [19, 37, 47, 49, 57–65]. Zeta size analysis showed Ag-NPs ranging from 1 to 500 nm, with size distributions consistent with green synthesis methods [35, 64, 66, 67]. SEM confirmed spherical particles with sizes of 30.5 nm, 33.8 nm, and 50.3 nm for different samples, aligning with previous findings using various plant extracts [37, 46, 68]. The zeta potential of nanoparticles plays a crucial role in their stability, with values indicating strong electrostatic repulsion that prevents agglomeration. As reported in previous studies, nanoparticles with a higher absolute zeta potential demonstrate better dispersion and enhanced stability, ensuring consistent and sustained antifungal activity [69–71]. The positive zeta potential observed in Ag-NPs, similar to the observations made with ZnS-based nanoparticles [71], suggests effective stabilization, preventing aggregation and enhancing their interaction with fungal cells for improved control of plant pathogens.

The antifungal potential of Ag-NPs against *C. capsici* was evaluated using poison food method, detached fruit assay, and leaflet assay. Ag-NPs significantly inhibited mycelial growth, particularly at 50 ppm, and were effective in curative applications. These findings align with previous studies demonstrating the antifungal properties of plant-based Ag-NPs [7, 19, 36, 37, 43, 68, 72]. Previous studies have shown that the antifungal mechanism of Ag-NPs involves disrupting multiple cellular components. Ag-NPs can severely damage the fungal cell wall and compromise the integrity of the cell membrane, leading to morphological changes such as wrinkling and depression of spores and mycelia [73–75]. This mechanism reduces mycelial respiration, inactivates key enzymes involved in the respiratory chain, and ultimately causes cell lysis, with cells twisting, expanding, and withering. Similarly, the mechanism of zinc sulphide–meerschaum nano bio-matrix (nZnS-MR) nanoparticles involves disrupting the fungal hyphal membrane, causing depolarization, protein leakage, and a morphological shift from cylindrical to de-turgid, shrunken, and ribbon-like structures [18, 76]. These findings elucidate the multifaceted mechanistic role of nanoparticles in suppressing phytopathogenic fungi, highlighting their potential as effective agents in plant disease management.

The results also highlighted the superior efficacy of curative over protective applications of Ag-NPs, agreeing findings from recent studies on various plant pathogens [39, 77–79]. In-vitro leaflet assays confirmed the inhibition of fungal infection and reduction of lesion size, consistent with previous research on the antifungal activity of Ag-NPs against various pathogens [80–82].

## Conclusions

This study highlights the efficient, eco-friendly synthesis of Ag-NPs using *P. guajava* leaf extract, offering a sustainable alternative for managing chilli anthracnose. The Ag-NPs demonstrated significant antifungal activity at low concentrations, suggesting their potential as a viable replacement for chemical fungicides. Future research should focus on optimizing the synthesis process for large-scale production and further investigating the long-term effects and environmental impact of Ag-NPs. Additionally, exploring the molecular mechanisms of their antifungal action and evaluating their effectiveness in diverse agricultural settings will be essential for their practical application in plant protection.

## Electronic supplementary material

Below is the link to the electronic supplementary material.

Supplementary Material 1

## Data Availability

All data generated or analysed during this study are included in this published article and its supplementary information files.
