# Supplementary material for: Green synthesis of silver nanoparticles using guava leaves: an effective strategy to control chilli fruit rot disease
Source: BMC Plant Biol. 2025 Apr 21;25:499. doi: 10.1186/s12870-025-06528-4 (PMC12010574; doi:10.1186/s12870-025-06528-4)
Supplement: Supplementary file 1 — Supplementary Material 1 [file 12870_2025_6528_MOESM1_ESM.docx]

| **Sr. no** | **Diffraction plane** | **Peak position (2θ)** | **FWHM (2θ)** | $\boldsymbol{\beta}\cos\boldsymbol{\theta}$ | **Particle size (nm)** |
| --- | --- | --- | --- | --- | --- |
| 1 | 200 | 44.49 | 0.1758 | 0.00283 | 54.4 |
| 2 | 220 | 63.61 | 0.25579 | 0.00379 | 40.6 |
| 3 | 311 | 77.71 | 0.34812 | 0.00472 | 32.4 |

**Table S1:** XRD analysis of S-NPs through Debye-Scherrer equation.

**Table S2:** FTIR analysis of biosynthesized S-NPs from *Psidium guajava* extract of S1.

| **Sr. No** | **Wavelength cm^-1^** | **Vibration type** | **Compound group** |
| --- | --- | --- | --- |
| 1 | 659.6 | C-C/C-H stretching | Aromatic benzene group |
| 2 | 818.4 | C-H/C-O/C-S stretching | Aromatic rings |
| 3 | 1025.32 | O-H/C-N/C-O stretching | Phenol/Alcohol/Ester/Ether group |
|  |  | C═C stretching | Alkenes |
| 4 | 1315.65 | O-H stretching | Alcohol/Phenols |
| 5 | 1363.77 | C-H/C-O bending | Alkyl ketones |
|  |  | N═O bending/N-O stretching | Nitric group |
| 6 | 1619.61 | C═O stretching | Carboxylic acid |
|  |  | C═C stretching | Ketone/Alkene/Aromatic ring |
|  |  | N-H bending | Carbonyl amide |
| 7 | 2849.1 | Sym C-H stretching | Alkanes |
| 8 | 2925.29 | O-H stretching | Phenols/Alcohols |
|  |  | Sym/Asym C-H stretching | Alkanes |
| 9 | 3277.38 | O-H stretching | Phenols/Alcohols |
| 10 | 3374.42 | C-H stretching | Alkyne |
|  |  | N-H stretching | Aliphatic amines |

**Table S3:** FTIR analysis of biosynthesized S-NPs from *Psidium guajava* extract of S2.

| **Sr. No** | **Wavelength cm^-1^** | **Vibration type** | **Compound group** |
| --- | --- | --- | --- |
| 1 | 652.38 | C-C/C-H stretching | Aromatic benzene group |
| 2 | 818.4 | C-H/C-O/C-S stretching | Aliphatic group |
| 3 | 1004.46 | C═C bending | Alkenes |
| 4 | 1087.87 | O-H/C-N/C-O stretching | Phenol/Alcohol/Ester/Ether group |
| 5 | 1184.12 | O-H/ C-N stretching | Alkyl amine group |
|  |  | Asym C-O stretching | Alcohol/Ester/Ether group |
|  |  | C═C stretching | Alkenes |
| 6 | 1308.43 | O-H stretching | Alcohol/Phenols |
| 7 | 1363.77 | C-H/C-O bending | Alkyl ketones |
|  |  | N═O bending/N-O stretching | Nitric group |
| 8 | 1619.61 | C═O stretching | Carboxylic acid |
|  |  | C═C stretching | Ketone/Alkene/Aromatic ring |
|  |  | N-H stretching | Carbonyl amides |
| 9 | 2926.66 | O-H stretching | Phenols/Alcohols |
|  |  | Sym/Asym C-H stretching | Alkanes |
| 10 | 3360.79 | O-H stretching | Phenols/Alcohols |
|  |  | C-H stretching | Alkyne |
|  |  | O-H stretching | Amines |

**Table S4:** FTIR analysis of biosynthesized S-NPs from *Psidium guajava* extract of S3.

| **Sr. No** | **Wavelength cm^-1^** | **Vibration type** | **Compound group** |
| --- | --- | --- | --- |
| 1 | 666.01 | C-C/C-H stretching | Aromatic benzene group |
| 2 | 832.03 | C-H/C-O/C-S stretching | Aliphatic group |
| 3 | 1004.46 | C═C bending | Alkenes |
| 4 | 1087.87 | O-H/C-N/C-O stretching | Phenol/Alcohol/Ester/Ether group |
| 5 | 1184.12 | O-H stretching | Alkyl amine group |
|  |  | C-N stretching | Amines group |
|  |  | Asym C-O stretching | Alcohol/Ester/Ether group |
| 6 | 1350.13 | C═C stretching | Alkenes |
|  |  | O-H stretching | Alcohol/Phenols |
|  |  | C-H/C-O bending | Alkyl ketones |
|  |  | N═O bending/N-O stretching | Nitric group |
| 7 | 1543.42 | Stretching vibration of proteins | Amides |
| 8 | 1626.83 | C═O stretching | Carboxylic acid |
|  |  | C═C stretching | Ketone/Alkene/Aromatic ring |
|  |  | N-H stretching | Carbonyl amide |
| 9 | 2856 | Sym C-H stretching | Alkanes |
| 10 | 2924 | O-H stretching | Phenols/Alcohols |
|  |  | Sym/Asym C-H stretching | Alkanes |
| 11 | 3353.57 | O-H stretching | Phenols/Alcohols |
|  |  | C-H stretching | Alkyne |
|  |  | O-H stretching | Amines |

**Table S5:** FTIR analysis of biosynthesized S-NPs from *Psidium guajava* extract of S4.

| **Sr. No** | **Wavelength cm^-1^** | **Vibration type** | **Compound group** |
| --- | --- | --- | --- |
| 1 | 652.38 | C-C/C-H stretching | Aromatic benzene group |
| 2 | 824.81 | C-H/C-O/C-S stretching | Aliphatic group |
| 3 | 1011.68 | C═C bending | Alkenes |
| 4 | 1087.87 | O-H/C-N/C-O stretching | Phenol/Alcohol/Ester/Ether group |
| 5 | 1191.33 | O-H stretching | Alkyl amine group |
|  |  | C-N stretching | Amines group |
|  |  | Asym C-O stretching | Alcohol/Ester/Ether group |
|  |  | C═C stretching | Alkenes |
| 6 | 1321.33 | O-H stretching | Alcohol/Phenols |
| 7 | 1363.77 | C-H/C-O bending | Alkyl ketones |
|  |  | N═O bending/N-O stretching | Nitric group |
| 8 | 1640.46 | C═O stretching | Carboxylic acid |
|  |  | C═C stretching | Ketone/Alkene/Aromatic ring |
|  |  | N-H stretching | Carbonyl amide |
| 9 | 2850.66 | Sym C-H stretching | Alkanes |
| 10 | 2925.33 | O-H stretching | Phenols/Alcohols |
|  |  | Sym/Asym C-H stretching | Alkanes |
| 11 | 3360.79 | O-H stretching | Phenols/Alcohols |
|  |  | C-H stretching | Alkyne |
|  |  | O-H stretching | Amines |

**Table S6:** FTIR analysis of biosynthesized S-NPs from *Psidium guajava* extract of S5.

| **Sr. No** | **Wavelength cm^-1^** | **Vibration type** | **Compound group** |
| --- | --- | --- | --- |
| 1 | 666.01 | C-C/C-H stretching | Aromatic benzene group |
| 2 | 818.4 | C-H/C-O/C-S stretching | Aliphatic group |
| 3 | 1011.68 | C═C bending | Alkenes |
| 4 | 1087.87 | O-H/C-N/C-O stretching | Phenol/Alcohol/Ester/Ether group |
| 5 | 1191.33 | O-H stretching | Alkyl amine group |
|  |  | C-N stretching | Amines group |
|  |  | Asym C-O stretching | Alcohol/Ester/Ether group |
|  |  | C═C stretching | Alkenes |
| 6 | 1315.65 | O-H stretching | Alcohol/Phenols |
| 7 | 1357.35 | C-H/C-O bending | Alkyl ketones |
|  |  | N═O bending/N-O stretching | Nitric group |
| 8 | 1626.83 | C═O stretching | Carboxylic acid |
|  |  | C═C stretching | Ketone/Alkene/Aromatic ring |
|  |  | N-H stretching | Carbonyl amide |
| 9 | 2852 | Sym C-H stretching | Alkanes |
| 10 | 2926.66 | O-H stretching | Phenols/Alcohols |
|  |  | Sym/Asym C-H stretching | Alkanes |
| 11 | 3353.57 | O-H stretching | Phenols/Alcohols |
|  |  | C-H stretching | Alkyne |
|  |  | O-H stretching | Amines |
